# Beyond quality improvement: exploring why primary care teams engage in a voluntary audit and feedback program

**DOI:** 10.1186/s12913-017-2765-3

**Published:** 2017-12-02

**Authors:** Daniel J. Wagner, Janet Durbin, Jan Barnsley, Noah M. Ivers

**Affiliations:** 1 Department of Community Health Sciences, Cumming School of Medicine, University of Calgary, 3280 Hospital Drive NW, Calgary, AB T2N 4Z6 Canada; 2 Centre for Addiction and Mental Health, 33 Russell Street, Toronto, M5S 2S1 Canada; 3 Department of Psychiatry, University of Toronto, Toronto, ON Canada; 4 Institute of Health Policy, Management and Evaluation, University of Toronto, Suite 425, 155 College Street, Toronto, ON M5T 3M6 Canada; 5 Family Practice Health Centre, Institute for Health Systems Solutions and Women’s College Hospital Research Institute, Women’s College Hospital, 76 Grenville Street, Toronto, M5S 1B2 Canada; 6 Department of Family and Community Medicine, University of Toronto, Toronto, ON Canada

**Keywords:** Audit and feedback, Primary care, Motivation, Implementation, Quality improvement, Organization of Care, Performance measurement, Clinician engagement

## Abstract

**Background:**

Despite its popularity, the effectiveness of audit and feedback in support quality improvement efforts is mixed. While audit and feedback-related research efforts have investigated issues relating to feedback design and delivery, little attention has been directed towards factors which motivate interest and engagement with feedback interventions. This study explored the motivating factors that drove primary care teams to participate in a voluntary audit and feedback initiative.

**Methods:**

Interviews were conducted with leaders of primary care teams who had participated in at least one iteration of the audit and feedback program. This intervention was developed by an organization which advocates for high-quality, team-based primary care in Ontario, Canada. Interview transcripts were coded using the Consolidated Framework for Implementation Research and the resulting framework was analyzed inductively to generate key themes.

**Results:**

Interviews were completed with 25 individuals from 18 primary care teams across Ontario. The majority were Executive Directors (14), Physician leaders (3) and support staff for Quality Improvement (4). A range of motivations for participating in the audit and feedback program beyond quality improvement were emphasized. Primarily, informants believed that the program would eventually become a best-in-class audit and feedback initiative. This reflected concerns regarding existing initiatives in terms of the intervention components and intentions as well as the perception that an initiative *by primary care, for primary care* would better reflect their own goals and better support desired patient outcomes. Key enablers included perceived obligations to engage and provision of support for the work involved. No teams cited an evidence base for A&F as a motivating factor for participation.

**Conclusions:**

A range of motivating factors, beyond quality improvement, contributed to participation in the audit and feedback program. Findings from this study highlight that efforts to understand how and when the intervention works best cannot be limited to factors within developers’ control. Clinical teams may more readily engage with initiatives with the potential to address their own long-term system goals. Aligning motivations for participation with the goals of the audit and feedback initiative may facilitate both engagement and impact.

**Electronic supplementary material:**

The online version of this article (10.1186/s12913-017-2765-3) contains supplementary material, which is available to authorized users.

## Background

Audit and Feedback (A&F), involving the provision of a summary of clinical performance over a specified period of time to healthcare providers, is a common quality improvement strategy [1, 2]. It is intended to support professionals or organizations in addressing gaps between ideal and actual care. If implemented successfully, A&F should be characterized as a feedback loop representing an iterative, self-regulating process [3]. Evidence from the most recent Cochrane Review found mixed effects; A&F led to a median improvement in process measures of 4.3% with an inter-quartile range between 0.5% and 16% for many clinical conditions and settings [1]. Subsequently, there has been a call for greater understanding regarding how and when A&F works best [2, 4].

Much of the literature examining how to optimize the impact of A&F has emphasised feedback design, possibly because this is the element over which organizations and researchers have the most control [5]. However, this focus on feedback design supports the implicit assumption that healthcare providers and organizations participate in A&F for the purposes of quality improvement and have no other motivations. There is little evidence to support this assumption; in Ontario, Canada, only a minority of primary care physicians access dashboards or practice reports that are available to them [6, 7]. Additional evidence suggests low uptake of A&F in other jurisdictions. For example, Trietsch et al. conducted a pragmatic cluster-randomized trial of an A&F intervention to reduce inappropriate prescribing behaviour by Primary Care Physicians in the Netherlands. Results from the study indicated that exposure to the A&F intervention led to no difference in prescribing patterns. In discussing their results, the authors suggested sub-optimal engagement with the feedback may have been due to lack of confidence in the intervention and a lack of motivation to realistically support the QI process [8]. In light of this evidence, other motivators to participation must also be considered in efforts to optimize A&F to support quality improvement activities.

If the end goal is to learn what works, where and in what context, it is necessary to understand the reasons why practitioners and organizations might (or might not) fully engage in A&F [4, 9, 10]. Unfortunately, little attention has been paid to factors that predict participation, interaction, or engagement with feedback interventions. The present paper aims to address this research gap by exploring the motivations of primary care practices for participating in a voluntary A&F program.

## Methods

### Setting and context

In 2004, the Government of Ontario introduced the Family Health Team (FHT) practice model, where a multi-disciplinary team of health service providers work together to provide high-quality, patient-centred care [11, 12]. In addition to physicians, FHTs also employ Nurse Practitioners, Social Workers and Occupational Therapists, among others. All providers are remunerated by the Ministry of Health and Long Term Care (MoHLTC); there are no patient user-fees to access the physicians or the allied health professionals at a FHT. The FHT model was developed at a similar time as literature began to emerge elsewhere regarding the development of patient-centred medical homes and are thought to meet similar standards and requirements [11, 12].

### Intervention

Data-to-Decisions (D2D) is a voluntary audit and feedback program developed by the Association of Family Health Teams of Ontario (AFHTO). AFHTO is a not-for-profit advocacy association mandated to promote the delivery of high-quality primary health care in Ontario. In 2014, the D2D initiative was launched to support Family Health Team efforts to measure and improve the quality of care they deliver [13]. The present investigation is a sub-study of a larger developmental evaluation commissioned by AFHTO to understand and improve D2D. While AFHTO facilitated this study by supporting recruitment and providing background information, the funding, design, analyses, and write-up were conducted independently from the association.

D2D provides a summary of a participating practice’s performance relative to peers. In D2D, a peer FHT is defined by four characteristics: Setting (Urban vs Rural), Teaching Status (None, Academic, Non-Academic), Access to Hospital Discharge Data (Yes, No), and Roster Size. The measures included in D2D are intended to be meaningful indicators of quality in primary care, guided by the seminal research of Barbara Starfield [14, 15]. D2D includes data from three primary sources: electronic medical records, annual patient experience surveys, and a primary care group practice report produced by Health Quality Ontario which is based on provincial administrative data holdings [6]. Through consultation with stakeholders, selected measures are incorporated into D2D from each source. Additional file 1 contains a summary of the Core Measures of D2D.

To support FHT quality improvement activities, including D2D, AFHTO collaborates with Quality Improvement Decision Support Specialists (QIDSS) located within FHTs. A QIDSS is a shared resource among a group of FHTs (known as a QIDSS partnership) to support data extraction, information production and ongoing analysis. This role is external to each FHT and is funded by the Ontario MoHLTC. AFHTO provides specialized training to QIDSS so they may facilitate and support FHT participation in D2D. However, availability of the QIDSS resource is not dependent on a FHTs participation in D2D.

The QIDSS is generally responsible for the audit component of the D2D program. Due to the multi-sourced nature of D2D, a data submission form is made available to participating FHTs. For FHTs with a QIDSS, this individual will collect the data from one of the four sources identified above for each measure and transcribe the value into the relevant field of the data submission form. FHTs without a QIDSS have an internal staff member responsible for this. For EMR data, AFHTO promotes the use of standardized queries which can be used by the individual auditing the EMR to ensure standardized reporting across participating teams.

### Data collection

Informants from FHTs were invited to participate in semi-structured interviews about their experience with D2D. Family Health Teams were eligible for this study if they had participated in at least one D2D audit and feedback cycle and had agreed to participate in the developmental evaluation of D2D conducted by AFHTO. Informants of interest were Executive Directors of FHTs as they were the intended recipient of the D2D feedback. At the discretion of the Executive Director, additional informants familiar with the FHT’s quality improvement activity (including D2D) were recruited to participate in the interview. To recruit specific teams for interview, criterion sampling was utilized to ensure variability with respect to practice Setting, Roster Size, Teaching Status, as well as the respective Standardized Adjusted Clinical Group Morbidity Index (SAMI; to indicate the complexity of patients rostered to a Family Health Team [16]). Invitations to participate in interviews were sent by email from AFHTO to the Executive Directors of identified FHTs. Rather than contacting all FHTs simultaneously, emails were sent to groups of twenty. The first group of invited FHTs were selected by AFHTO, independently of the research team. However, each subsequent invitation was sent to a group of FHTs selected by the lead investigator (D.W.) to ensure representation across the four measures described above. Further details about the measures used to monitor recruitment can be found in Additional file 1.

The Consolidated Framework for Implementation Research (CFIR) was used as the conceptual framework for this study. CFIR encompasses a range of concepts that are applicable to a wide variety of contexts in evaluating the implementation of interventions [17, 18]. In its development, the framework consolidated theories across implementation science to yield a composition of 39 constructs (i.e. relative advantage, peer pressure, readiness for implementation, etc.), which are grouped into five domains: Characteristics of the Intervention, Inner Setting, Outer Setting, Characteristics of Individuals and Process. The template guide available from CFIR developers [17, 18] was modified for the purposes of this study, piloted, and then further revised prior to recruitment to inform question sequencing [19].

The interviews were conducted by a single interviewer (D.W.) between March 1 and April 30, 2016. Each interview explored the rationale for participating in D2D, how each FHT used D2D, and the resources necessary to support participation. Throughout data collection, minor iterative revisions were applied to the interview guide to introduce additional probes, clarify wording and pursue emerging themes. While some questions may have been re-phrased, no content was removed from the interview guide and all content was meant to be consistent with the CFIR construct(s) which informed each particular question. Interviews were conducted at the time of the participants’ choosing either by telephone, Skype, or in-person at the offices of the Family Health Team. Interviews were audio recorded and transcribed to produce verbatim, electronic transcripts for qualitative analysis. Sampling continued until the authors were in agreement that saturation had been reached [20].

### Analysis

A framework approach was utilized to analyze the transcripts based on constructs from the CFIR framework. These were used to populate the initial version of the codebook used for the analysis [19]. Double coding was performed on three transcripts by the lead investigator (D.W.) and another author (J.D.) to validate construct assignment. Each transcript was selected at random, using a random number generator based on the identification number of each interview. Both reviewers coded one transcript independently and compared the results. If a reviewer felt that a code did not match any construct, it was labelled as “other”. These were then discussed by both D.W. and J.D. to determine if any CFIR constructs would apply to the selected text. If no CFIR constructs were applicable, a new non-CFIR code was defined and included in the codebook following consultation with the remaining investigators (J.B. and N.I.). Only one such code resulted from this process: *Parallel Initiatives.* This was defined as an intervention occurring simultaneously which shared characteristics with the intervention being implemented. The updated codebook was then used to code the second, and third transcripts. Following this procedure, the remaining transcripts were single-coded by the lead investigator.

To analyze data for this investigation, a framework was constructed using CFIR constructs which were identified a priori to the analysis, but following data collection, as factors which might influence participation in D2D. Specific constructs included: *Relative Advantage, Evidence Strength & Quality, Peer Pressure, External Policy and Incentives, Tension for Change, Leadership Engagement, Opinion Leaders,* and *External Change Agent.* The generated framework was then analyzed inductively to identify emergent themes from the data. For reporting purposes, themes were stratified into one of three domains (Intervention Characteristic, Outer Setting, Inner Setting) from which the respective CFIR construct belonged. Qualitative analysis was supported with the use of the NVivo software application for Windows [21]. Descriptive analysis of practices invited for interviews and interview characteristics were performed using the R statistical software program for Windows [22].

## Results

A total of 118 Family Health Teams were eligible to participate in interviews. Of those, 45 were invited to participate with 21 expressing initial interest. Thematic saturation was reached after interviews with 25 key informants from 18 Family Health Teams across Ontario. Table 1 includes descriptive statistics comparing FHTs which were invited, expressed interest and were interviewed on the variables used to inform recruitment. The interview sample contained representation from rural and urban, FHTs, those with and without access to hospital discharge data, and different teaching status. Little variability was observed between the interviewed and non-interviewed samples with respect to roster size and SAMI. Interviews lasted a mean of 53 min (SD = 8 min) ranging from 35 to 63 min. The Executive Director was usually involved, but number and type of informants differed within each interview as different practices had different leaders for the D2D initiative.

**Table 1:** Characteristics of family health teams and interview formats

FHT characteristics				
	Total	Recruited	Not Interviewed	Interviewed
Sample Size	118	45	101	17^1^
	Mean (SD)	Mean (SD)	Mean (SD)	Mean (SD)
Roster Size	20,788 (32,419.3)	19,417 (17,383.3)	20,994 (34,644.8)	19,622 (15,126.5)
SAMI	0.99 (0.1)	0.99 (0.1)	0.99 (0.1)	1.00 (0.1)
	% (n)	% (n)	% (n)	% (n)
Setting				
Rural	47 (56)	42 (19)	50 (51)	29 (5)
Urban	53 (62)	58 (26)	50 (50)	71 (12)
Hosp. Discharge Data	61 (72)	69 (31)	59 (60)	71 (12)
Teaching Status				
Academic	17 (20)	9 (4)	18 (18)	12 (2)
Non-Teaching	26 (31)	33 (15)	24 (24)	41 (7)
Teaching	57 (67)	58 (26)	58 (59)	47 (8)
Interview Formats				
	1-on-1% (n)	2-on-1% (n)	3-on-1% (n)	Total % (n)
By Practice	66.7 (12)	27.8 (5)	5.6 (1)	100 (18)
Participant Type				
ED^2^	71.4 (10)	21.4 (3)	7.1 (1)	100 (14)
MD^3^	0.0 (0)	66.7 (2)	33.3 (1)	100 (3)
QIDSS^4^	25.0 (1)	50.0 (2)	25.0 (1)	100 (4)
IHP^5^	0.0 (0)	100 (1)	0.0 (0)	100 (1)
Other	33.3 (1)	66.7 (2)	0.0 (0)	100 (3)
Total	48.0 (12)	40.0 (10)	12.0 (3)	100 (25)

Notes: One interview was held with a QIDSS alone. Given that these staff work with multiple FHTs (in this case 4), these practices were excluded from this summary

ED = Executive Director

MD = Physician Leader at the Family Health Team

QIDSS = Quality Improvement Decision Support Specialist

IHP = Interdisciplinary Health Professional (Nurse, Dietician, Social Worker, etc.)

SAMI = Standardized Adjusted Clinical Group Morbidity Index

A summary of each finding stratified by CFIR domain is presented with a sample quote in Table 2. Supporting quotations for each result are included in the corresponding Box following each result.

**Table 2:** Summary of motivations to participate in a voluntary audit and feedback intervention by CFIR domain

Theme	Summary
Outer Setting	
Policy Advocacy	D2D was identified as a vehicle to support two policy priorities: 1) The future direction of Primary Care quality improvement and performance measurement; and 2) The value and contribution of FHTs to Ontario’s Health Care System.
*“We wanted to be able to work with AFHTO to start being able to direct where Health Quality Ontario was asking us to go on our quality improvement by using data that was more accurate or more up to date, to create those conversations…” (ID = 015)*	
Peer Influence	Knowledge of peer participation in D2D facilitated participation in a minority of cases.
*“Other people participating doesn’t really drive our D2D work…”(ID = 018)*	
Good Soldier Phenomenon	Observed in two ways: 1) Some informants had external responsibilities to AFHTO. Participation in D2D was part of their efforts to support AFHTO initiatives. 2) Some FHTs agreed to participate to fulfill their responsibility as a member of AFHTO.
*“…we participate, really to be good corporate citizens.” (ID = 001)*	
Inner Setting	
Availability of Implementation Leader	A dedicated staff person to support implementation was seen as essential component for participation. Without this resource, most practices interviewed would not participate.
*“…our [QIDSS] really kind of pushed it too, and he was there to help us get the information. That made it a bit easier.” (ID = 015)*	
Development of QI Capacity	D2D was viewed as a means to improve teams’ development of quality improvement capacity.
*“We wanted to be able to measure how we’re doing, to be able to compare ourselves with similar groups throughout the province, but knowing that, for us, we were just starting the measurement process. And we wanted to know what we’re able to do and what our limitations were.” (ID = 012)*	
Intervention Characteristics	
Promise of Future Potential	Participation was influenced by a promise of a “best-in-class” data tool which will be developed by ongoing participation. Desirable features included: Peer comparison and Benchmarking, the use of up to date data, the consolidation of data from a variety of sources and that the tool would be directly informed by participant feedback.
*“…I think that need is right now mostly based on a promise. The promise is what is going to happen with future iterations, and that its going to continue to develop until it actually is a robust, useful, accessible, meaningful exercise. I think we’ve taken initial steps towards that, but we need it to continue in that area” (ID = 014)*	
*Evidence Base*	No FHTs considered an evidence base in deciding to participate in D2D.
*“I don’t think I have to go to my IT expert and say, do you think measuring how we’re doing is a good idea? It just kind of is. I don’t know how else to say it. I never presented to the group what was the evidence base behind D2D. To me…this is good for QI, this is good for accountability…” (ID = 017)*	

### Outer setting

#### Policy advocacy

Participants emphasized the role of D2D in AFHTO’s policy advocacy efforts as a motivator for their participation. They believed that D2D could be used to advocate on the future direction of primary care performance measurement. FHTs were concerned that the quality indicators within other feedback reports were not representative of their true performance and did not support better patient care or outcomes. The potential to use D2D to advocate regarding the value of the FHT practice model to the government and the public was also appealing to participants (Table 3).

**Table 3:** Quotations on policy advocacy

• AFHTO has been very engaged and very involved. They have pushed because they knew there was a gap there. And they solved that for our organization, so they need … That involved from AFHTO is really, really important. Even if this is something that is taken over by the ministry, I think having them involved as speaking on behalf of the family health teams is really, really important. (ID = 012)
• Getting back to what I was saying before, I’d rather be leading the way than told what to do and how to do it. So I see D2D as our opportunity to really put it out there and say look, to the government, if you’re going to try to measure how well we’re doing and the quality of our healthcare, I’d rather be the one saying this is a known shown evidence-based way to do it. I think we’re working on that with D2D. (ID = 014)
• I need a need for D2D, I think it depends on what the raison d’etre of D2D is. My understanding, perhaps incorrect, initially, was that it was clear that there was going to be reporting mechanisms being put in place, being forced upon us from the ministry, and that AFHTO wanted to try to get in on the ground floor to try to see … basically to influence the ministry. What’s feasible, what’s reasonable, what’s important to primary care, and to get clinicians involved in trying to influence those decisions. I think from that respect, D2D is important. (ID = 001)
• The other part of it was we wanted to be able to work with AFHTO to start being able to direct where Health Quality Ontario was asking us to go on our quality improvement by using data that was more accurate or more up to date, to create those conversations between AFHTO and HQO. (ID = 015)
• Well, I think it puts us in a good position if indicators that we report on D2D are ones expected of us, say from the Ministry. I think that puts us in a great position because we’re already able to report on them. (ID = 006)
• Knowing that we were in a climate where primary care, just in general, was being scrutinized around performance and whether or not it was actually making an impact, patient-centred and outcomes and things like that. It was always, from the get-go, probably, the underlying reason why we wanted to participate in the day-to-day because we wanted to tell the story. Yes, don’t believe everything you are hearing out there. At the local level, we are making a lot of really good progress around patient outcomes. (ID = 003)
• So sometimes perhaps the Ministry will be looking for information that doesn’t paint the true picture of what primary care, the members of AFHTO are doing. So by having the association work with us to generate these measurement reports, we can actually give them to the Ministry at different levels of how that goes and share the information and say this is where we’re doing very well, this is where we’re making a difference. You may not be aware of that based on what you’re looking to collect yourselves. So that’s my thought on it. (ID = 019)
• I know there’s been a lot of question regarding the impact the family health teams have had. It was a rather expensive rollout for the government to establish all the teams, and they’ve obviously put a pause on the expansion of those teams over the last year or so, they’ve slowed down quite a bit, actually. (ID = 021)
• I would argue that people didn’t view this so much as a policy climate as they viewed it as a political climate where the Ministry was trying to justify the huge price tag of family health teams. And being able to demonstrate a high level of performance impact on the health care system, which I understand, I think that was fine. I think, initially, people viewed this is an overwhelming experience. (ID = 005)

#### Peer influence

Only a small number of teams cited peer influence as a facilitator to their participation. In these cases, participants acknowledged that their awareness of their peers’ participation served as a catalyst to join the D2D A&F initiative. The rest of the participants challenged the role of peer influence – suggesting they just thought it would be a good idea. Interestingly, some Family Health Teams perceived that they would specifically influence other teams to participate in the initiative (Table 4).

**Table 4:** Quotations describing peer influence

• I think we took the plunge regardless early in the first reiteration of D2D because we just thought it was the right thing to do. We didn’t really know what would come out of it exactly so I don’t know how much influence knowing others were participating, but we thought it was safe enough for us to, as I would say, dip your big toe in, see what came back, and how many teams participated. I believe in early days it wasn’t a lot of teams, but I think other teams were convinced to join after the fact when we were able to share some of our results. So maybe we influenced other teams, but I don’t know if other teams influenced us. (ID = 014)
• M: are they supportive of D2D because they know other FHTs are participating? Does that factor in at all? R: No, I don’t think so. (ID = 016)
• M: At the very beginning, you mentioned that you were aware of other organizations participating in D2D. R: Yeah, other family health teams. M: Did this impact the support for D2D in your setting? R: Yes, it did. (ID = 008)
• Yes, other people participating doesn’t really drive our D2D work. It’s great, the more people share, the better idea we have of the benchmarking in terms of generalizing it. But, in terms of working with other people in the area, other organizations like ours, that really doesn’t matter, I don’t think, too much. (ID = 018)

#### Perceived obligations

The mechanism by which this was observed depended on whether a FHT leader held a leadership role at AFHTO. At the time of the interviews, five participants noted experience on the AFHTO board of directors. In this case, informants cited their leadership position at AFHTO as a facilitator to their practice’s participation. Informants uninvolved with AFHTO governance suggested that their practice’s participation in D2D was facilitated by the perception that it was an AFHTO membership requirement (Table 5).

**Table 5:** Quotations describing perceived obligations

• So I think we participate, really to be good, corporate citizens. I think AFHTO does good work, and if we can help them do that good work, then that’s why we’re doing it. (ID = 001)
• We just want to participate and be an active member for AFHTO. (ID = 007)
• I think, just as my bias, I am on the AFHTO board. So, to be part of the board, you need to be very, very supportive in the initiatives that come our way. (ID = 003)
• …we actually have some people with our FHT that have helped influence D2D. They’ve been asked to be on some steering committees and stuff. (ID = 017)
• Name-X, just so you know, and you probably do know already, Name-X is on the Board of AFHTO, and Name-X is on the Indicators Working Group. I’m on the Steering Committee, and I’m head of the DM Management Group so we do have a high-level view of this. (ID = 014)
• Well, it doesn’t hurt that I sit on of the indicator working group ... And so, I would have to say that we didn’t get a lot of pushback from our board when we suggested starting to get involved with this, because they probably want to support what their ED [executive director] and lead physicians are involved with. (ID = 002)

### Inner setting

#### Availability of implementation champion

A significant facilitator in deciding to participate in D2D was the availability of resource to conduct the D2D audit within the practice. Except for a few cases, this role was filled by a Quality Improvement Decision Support Specialist (QIDSS) who collated the data from the three sources to enable participation (Table 6).

**Table 6:** Availability of implementation leader

• Really, it started with the leadership from our Quality Improvement Decision Support Specialists in terms of their working relationship with AFHTO. They were really the cheerleaders for D2D and trying to fan that out across the Family Health Teams that they support, promoting the value of using D2D and encouraging people to contribute data. (ID = 018)
• The executive directors of those nine FHTs, we meet once a month, and these are the sort of things we discuss at those meetings. It really was with … Our quality decision support specialist, really kind of pushed it too, and he was there to help us get the information. That made it a bit easier. (ID = 015)
• Well, no, at first I was always curious, but we didn’t have anybody. We had a lot of turnover in our staff, so it would have been me, and that’s really not my role. So, it wasn’t until Name-X came on board, our QIDSS specialist, he’s extremely helpful in guiding us and supporting us in his role. So, that’s when we started to … he’d come up and spend a week with us. So, that’s when we said, okay, if we’re going to take part, we should take part and we have this assistance. He’s kind of guiding us. (ID = 008)

#### Development of QI capacity

A common internal factor among FHTs which facilitated the decision to participate in D2D was the need to develop their capacity for quality improvement. Informants revealed that many FHTs lacked any formal QI processes or committees prior to D2D. The structured approach of D2D to support their own QI efforts was appealing to FHTs as they could use it as is, and be supported by the larger FHT community. This approach was preferable to developing an A&F program independently (Table 7).

**Table 7:** Development of QI capacity

• It’s hard to say because we didn’t really have quality improvement in place before. So, it’s been a great vehicle to advance it. So, at the time, when our organization was old enough to be able to start thinking about quality improvement, that is when it started. So, we could vote as a team. So, we have really grown up with it. So, it’s, maybe, been a consolidating focus. (ID = 013)
• When we started to involve ourselves in quality improvement initiatives in a more formal capacity, we recognized the need to, I guess, retool the organization in terms of training, in terms of resources, to be able to do it properly. We didn’t have the internal expertise to do that. When we were able to hire QIDSS as an improvement decision support specialist that was very helpful, but still having more of a formal process in place that would allow us to structure and tap into data in a more meaningful way was helpful. D2D seemed to provide a method for us to be able to do that, and one that was aligned with more of a provincial initiative as well, so we didn’t feel like we were doing this on our own, but in fact was part of a larger quality improvement community that was engaged. (ID = 005)
• We wanted to participate because we knew the importance of being able to measure the work that we’re doing and we didn’t know how to do this in a meaningful way. We didn’t know if we wanted to ... We thought the D2D would help support us, would help us look at what we are able to measure, what we’re struggling to measure, help us in ways that we’d be able to get that information and be able to compare it with the other family health teams across the province. We wanted to be able to measure how we’re doing, to be able to compare ourselves with other similar groups throughout the province, but knowing that, for us, we were just starting the measurement process. And we wanted to know what we’re able to do and what our limitations were. (ID = 012)

### Intervention characteristics

#### Promise of what the intervention will become

A key factor that drove teams to engage with the intervention was that they felt it had the potential to become a state-of-the-art initiative. Practices were interested in an A&F initiative that presented recent data, of measures perceived to be meaningful to their practices, and that captured the full-scope of primary care practice. Participants described the multi-source audit as appealing because it would collate all the performance information in one place. Another important driver for participation was the feature where practices are encouraged to provide feedback to AFHTO to improve the audit and feedback initiative. Having this final component helped the practices feel that they had a stake in their own measurement and increased engagement because it reinforced the idea that the intervention had the potential to incorporate desired features that would give it a relative advantage over other similar initiatives (Table 8).

**Table 8:** Quotations regarding how the promise of the initiative influenced participation

• Yes I do, and I think that that need is right now mostly based on a promise. The promise is what is going to happen with future iterations, and that its going to continue to develop until it actually is a robust, useful, accessible, meaningful exercise. I think we’ve taken initial steps towards that, but we need it to continue in that area. (ID = 014).
• And it’s a good thing to be involved because you’re getting data and you can compare it to the other teams in the area, whereas, really there isn’t a system out there that was doing that before D2D. (ID = 006).
• I think some of it was the data that we were getting from the Health Data Branch was so old that we wanted something that was more up to date and D2D was offering the ability to pull the more up to date data. Because Health Data Branch is always a year or two years behind. That really was part of it. (ID = 015).
• So, we don’t have any other quality person. So, I don’t have time to be combing through all these reports. So, D2D is the one-stop shop. (ID = 013).
• That said, though, D2D has that … unlike many other reports that are out there, it is very much about what the members want and what the members see as valuable (ID = 003).

#### Evidence base

No teams cited the evidence base for performance measurement and feedback as either a barrier or a facilitator in their decision to participate in the intervention. On the role of evidence in deciding to participate in the audit and feedback initiative, the majority of informants indicated they supported the principles of measurement and feedback and didn’t think a review of the evidence was necessary (Table 9).

**Table 9:** Quotations regarding the way in which the evidence base for A&F influenced participation

• I don’t know of any evidence that they put forward to except that they did mention Dr. Starfield as a … her research project about how she studies those three principles of cost, quality, and capacity. And so, there was that as a framework that they were using that I thought was brilliant as far as going that route, and using that as the principles of where they wanted to go with measuring for primary care … It helped me sell it within my own self, that we were on the right track. So I’d have to say, I’ve been excited about being involved with it because of those principles. (ID = 002).
• I don’t think I have to go to my IT expert and say, do you think measuring how we’re doing is a good idea? It just kind of is. I don’t know how else to say it. I never presented to the group what was the evidence base behind D2D. To me, it was like, this is good for QI, this is good for accountability. That makes sense to all of us, let’s go for it. (ID = 017).

## Discussion

Despite its varied effectiveness, A&F remains a popular approach to quality improvement. The present study identified several factors that motivated engagement with a voluntary A&F initiative developed by a party external to the practices (Table 2). Participating practices engaged with the initiative, in part, to contribute toward policy advocacy goals. They took part because they trusted the lead organization to act on their behalf, joining not necessarily because they thought the initiative would help them with improving quality of care but because they wanted to contribute as good soldiers toward a desired goal.

Implementation of A&F programs (i.e., engaging with and taking action on the data to change practices) may also be promoted through good soldiers, where developers would encourage Organizational Citizenship Behaviour (OCB) [23]. This is behaviour which is “discretionary, not directly or explicitly recognized by the formal reward system and that in the aggregate promotes the effective functioning of the organization” [23]. The process by which OCBs are encouraged, thereby pressuring agents to act as good soldiers is referred to as citizenship pressure [24]. A 2009 study exploring citizenship pressure found that employees who feel pressured to be good soldiers tend to engage in more OCBs. Thus, an external A&F developer could encourage its stakeholders also involved in practice administration to support A&F implementation. However, the study notes that citizenship pressure may have unintended consequences such as increased work-family conflict, job stress and intentions to quit. In the context of this study, this may be interpreted as too much citizenship pressure leading to risk of burnout and/or disengagement in the ongoing development of D2D [24, 25].

Many informants highlighted the opportunity D2D presented to develop capacity for practice-level quality improvement. However, the availability of the Quality Improvement Decision Support Specialist as the implementation leader was frequently cited as a requirement for their participation. Given that this agent is independent of the practice, it may be the case that their participation was a factor of convenience, rather than strategic need. This is demonstrated by the finding that the QIDSS resource was responsible for the collation of data from the different audit sources. A tension may exist in this practice environment whereby practices are aware of the need to develop capacity in QI, but lack the incentives to invest their own time and resources towards this effort [26]. Developers external to feedback recipients may wish to consider this scenario in managing their expectations for A&F implementation.

In the context of this investigation, the impact of peer pressure on practice motivation was less than anticipated. Some FHTs believed their participation would pressure others, while other FHTs suggested peer pressure played no role in their participation. The latter group of FHTs supported the idea of measurement to monitor performance but did not believe peer participation was an important factor in their engagement with the initiative. It is possible that other motivating factors play a more important role in participation. Alternatively, people may be unaware of the strong influence that social pressures can have [27]; those expressing a willingness to influence others suggests that their ongoing participation may be facilitated by a desire to be perceived as leaders. Further research is necessary to determine the extent to which peer pressure could motivate participation in externally developed A&F programs.

With respect to the evidence base, consensus emerged among informants that A&F was a common-sense approach to quality improvement. However, given the inconsistent effectiveness of A&F, it is striking that no practice considered the available evidence for different ways in which A&F might be used to generate better outcomes [28]. As D2D was an externally developed A&F initiative, practices may have assumed this work was completed by the developer. Alternatively, this oversight may have manifested from a lack of awareness regarding the large evidence base which exists regarding organization of care and health care management [29].

In the context of a voluntary A&F intervention such as D2D, participation can be considered a necessary and independent contributor to implementation. As described in the introduction, successful implementation should be characterized by a feedback loop representing an iterative, self-regulating process [3]. In the context of this feedback loop, participation should be viewed as an external decision node. This distinction is important in assessing the findings of the present study in the context of the subsequent implementation of the A&F program. At the time of this writing, the relationship between the motivators to participation and the subsequent implementation have not been explored. This should be the subject of future research. However, such efforts will be complex as the implementation of an A&F program is subject to a range of factors beyond motivations to participate – such as the 15 recommendations suggested by Brehaut et al. [28].

It may be difficult to disentangle factors which might influence implementation and motivations to participate in an A&F initiative. A 2011 study employed a grounded theory approach to explore if there were aspects of the A&F process that impact physicians’ acceptance of feedback and their practice behaviour. Results indicated that feedback which is timely and individualized would facilitate implementation [30]. Goldberg et al. developed a conceptual framework to explain the mechanisms of influence and contextual modifiers on performance measurement in physician practices [31]. Four key pressures in adoption were identified: incentives, organizational relationships, access to resources, and competing work demands. Leadership priorities and support as well as organizational culture were identified as moderators to the identified pressures [31].

The impact of these pressures and moderators are prevalent in other investigations of A&F in primary care and are further demonstrative of the disentanglement problem. For example, one study (conducted in a similar context to the present investigation) reported several physician-identified barriers to feedback use. These included data validity, the availability of resources to support QI, and balancing standardized quality targets with patient-centred care [32]. At a practice level, Johnston et al. observed that primary care teams were receptive of performance feedback and that team members thought that a feedback cycle could improve their organizational culture towards measurement and teamwork. However, findings also revealed that few teams or individuals understood how to use the data they received [33]. As a result, while many teams cited the promise of D2D’s future potential as a motivator to their participation, the same pressures and moderators may influence motivation to fully participate and eventually implement the A&F program.

Lastly, it is important to consider that motivations to participate in an A&F program may vary beyond those identified in the present study. Motivations to participate may be of less interest in mandatory A&F programs due to the absence of choice. Future research will need to explore the varying motivations to participate in voluntary A&F programs across practice settings. Of particular interest will be the exploration of motivations between A&F interventions which are core components of research projects and those which are independent of the research continuum. Given that D2D was developed and managed by AFHTO, a community based advocacy organization, findings from the present study may be classified into the latter category. As the findings of this study suggest, such efforts should elucidate the complexity of motivations to participate in voluntary A&F programs which extend beyond quality improvement.

### Limitations

Findings of this study should be understood in the context of four primary limitations. First, participants were early adopters of D2D and many had roles on the AFHTO board or in D2D’s development. As a result, the proportion of practices motivated to participate in D2D given their nature as “good soldiers” may be over represented. There may have been other factors beyond those identified which may have influenced FHTs beyond those interviewed to participate in the A&F program. Second, findings may not be generalizable to other primary care settings or jurisdictions. Further research is required to determine the range of factors that influence engagement with A&F interventions across contexts and practice types. Third, while the analysis was supported by double coding of interviews, thematic coding was done independently with no validation procedure. The impact of this limitation on the results is mitigated by the application of the framework methodology in concert with the use of deductive coding.

Fourth, while the use of a previously validated framework (CFIR) should support the prospective reproducibility of this investigation limitations persist. Primarily, the deductive approach to coding described in this study creates a risk that some aspects of implementation which are not in the framework are overlooked. This risk was acceptable to the research team in the context of the many strengths of the CFIR approach. In particular, it is an increasingly common well-established and well-evidenced framework that is thought to be fairly comprehensive. Moreover, use of the CFIR helps to ensure new studies build on the knowledge generated in preceding inquiries by using similar terminology for implementation related phenomenon.

## Conclusion

Audit and Feedback is a foundational quality improvement strategy and is a feature of many high performing health systems. However, health care providers (whether individuals or teams) do not always fully engage in these initiatives. This study identified several motivating factors, beyond using data to support local quality improvement, which facilitated participation by FHTs in a voluntary A&F intervention (Table 2). Practices chose to opt-in in large part to support the policy goals of the organization leading the initiative. Given that engagement with A&F is a key step in data-driven quality improvement, developers should be mindful to leverage motivations to promote participation and engagement in A&F programs. In turn this may facilitate long-term quality improvement as developers and practices use A&F to meet shared policy goals or other objectives. Given that the motivations elicited in the present study are by no means complete, further research is necessary to explore the motivations to participate in A&F in a range of jurisdictions and practice settings.

## Additional file

Additional file 1: D2D Core Measures. This file contains a table summarizing the core measures included in the D2D Audit and Feedback intervention. The data are meant to provide interested readers in additional information regarding the scope and measures included in the intervention of focus for this study. (DOCX 16 kb)
