# Supplementary material for: Beyond quality improvement: exploring why primary care teams engage in a voluntary audit and feedback program
Source: BMC Health Serv Res. 2017 Dec 2;17:803. doi: 10.1186/s12913-017-2765-3 (PMC5712172; doi:10.1186/s12913-017-2765-3)
Supplement: Additional file 1: — D2D Core Measures. This file contains a table summarizing the core measures included in the D2D Audit and Feedback intervention. The data are meant to provide interested readers in additional information regarding the scope and measures included in the intervention of focus for this study. (DOCX 16 kb) [file 12913_2017_2765_MOESM1_ESM.docx]

# Core Measures of the D2D Audit and Feedback Program

| **Variable** | **Definition** | **Source** |
| --- | --- | --- |
| Same/Next Day Appointments | Proportion of patients who reported they had an appointment on the same day or next day after contacting their family health team. | HQO^1^ Patient Experience Survey |
| Involved | Proportion of patients who felt involved in decisions about care and treatment. | HQO^1^ Patient Experience Survey |
| Reasonable wait for Appointment | Proportion of patients who indicated that they could book an appointment within a reasonable time. | HQO^1^ Patient Experience Survey |
| Rostered Visits | Percentage of total primary care visits that are made to the physician with whom the patient is rostered or virtually rostered. | ICES^2^ |
| Hospital Readmissions | Proportion of patients with an acute inpatient hospital stay for selected conditions who have a subsequent non-elective readmission within 30-days post discharge. | ICES^2^ |
| Colorectal Cancer Screening | The proportion of patients aged 50 to 74 years old with a fecal occult blood test within 24 months, a flexible sigmoidoscopy within 5 years, or a colonoscopy within 10 years of the report cut-off date. | ICES^2^ |
| Cervical Cancer Screening | The proportion of female patients, aged 21 to 69, who had a papanicolaou smear within the past three years. | ICES^2^ |
| Immunizations | Proportion of rostered 2-year-old children who are up-to-date for immunization coverage. | FHT-EMR |
| Practice Setting^3^ | Dichotomous variable referring to the type of community served – Urban or Rural | FHT |
| Teaching Status^3^ | Categorical value as “Academic”, “Teaching” and “Non-Teaching” based on a FHTs ongoing relationship with a medical school or other educational program. | FHT |
| Access to Hospital Discharge Data^3^ | Dichotomous variable referring to the FHT’s implementation of a service to update its EMR with Hospital discharge information. | FHT |
| Cost | Refers to the total healthcare system cost of patients rostered/served by the Family Health Team. These costs may include adjustments to reflect age, sex or complexity of patients. | ICES^2^ |
| Standardized Adjusted Clinical Group Morbidity Index (SAMI)^3^ | A surrogate measure of the complexity of patients served by each family health team. | ICES^2^ |
| Notes:   1. HQO = Health Quality Ontario. Is the advisor to the Government of Ontario on heath care quality. Activities include: Monitoring and reporting on system performance, guidance on important quality issues, evidence assessments, patient engagement among others. 2. ICES = Institute for Clinical and Evaluative Sciences. Houses much of Ontario’s inventory of coded and linkable healthcare data sets. This includes much of the publicly funded administrative heath service records for the Ontario population eligible for universal heath coverage since 1986. 3. These four measures were used to inform the recruitment procedure described in the methods section of the manuscript. | | |
